# Rex Shunt for Extra-Hepatic Portal Venous Obstruction in Children

**DOI:** 10.3390/children9020297

**Published:** 2022-02-21

**Authors:** Jinshan Zhang, Long Li

**Affiliations:** Department of General Surgery, Capital Institute of Pediatrics, Beijing 100020, China; zjs851@126.com

**Keywords:** extra-hepatic portal venous obstruction, portal hypertension, Rex shunt, children, treatment

## Abstract

Rex shunt, which was first put in use in 1992, has been considered as an ideal surgical method for the treatment of extra-hepatic portal venous obstruction (EHPVO) due to its reconstruction of the hepatopetal portal blood flow. However, despite its long tradition, there are only a few reports about the application and advances in Rex shunt for the treatment of EHPVO in children. In this paper, we summarized the literature related to Rex shunt and discussed the new advances of Rex shunt in the following aspects: surgical method of Rex shunt, the indications of Rex shunt, the strengths of Rex shunt, the effectiveness of Rex shunt, factors affecting the efficacy of Rex shunt, methods that improve the prognosis of Rex shunt, and treatment strategy for recurrence after Rex shunt.

## 1. Introduction

**Figure 1:**
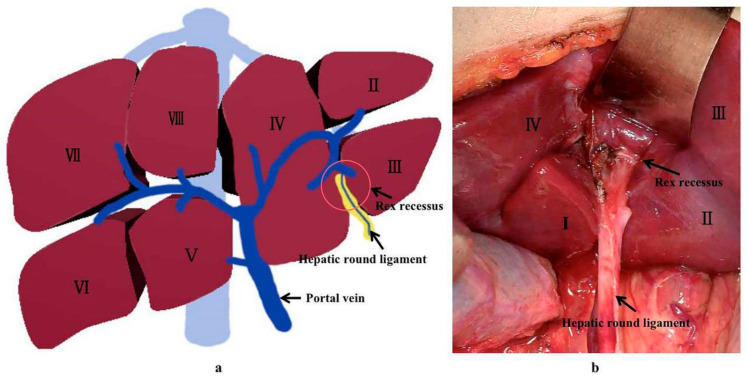
The location of Rex recessus ((**a**) a drawing picture; (**b**) an anatomic picture).

Extra-hepatic portal venous obstruction (EHPVO) is a condition that leads to prehepatic portal hypertension, where the forward hepatopetal flow of portal blood from the superior mesenteric vein, splenic vein, and coronary vein is impeded through a normal portal vein by a relative or complete obstruction [1]. Rex shunt was considered as an ideal surgical method for treating EHPVO due to its reconstruction of the portal blood flow into the liver [2]. Rex shunt refers to a surgery where the extra-hepatic portal blood is drained into the left branch of intra-hepatic portal vein in Rex recessus by bypassing or transposing a grafted vein, through which a hepatopetal portal blood is reconstructed. The word “Rex shunt” originated from the anatomic structure of Rex recessus (located between the liver segments III and IV and at the root of the hepatic round ligament) (Figure 1), in which the portal blood is re-shunted into the liver through the Rex shunt.

In this paper, we summarize the literature about Rex shunt and discuss the new advances of Rex in the following aspects: surgical method of Rex shunt, the indications of Rex shunt, the strengths of Rex shunt, the effectiveness of Rex shunt, factors affecting the efficacy of Rex shunt, methods improving the prognosis of Rex shunt, and treatment strategy for recurrence after Rex shunt.

## 2. Surgical Method of Rex Shunt

**Figure 2:**
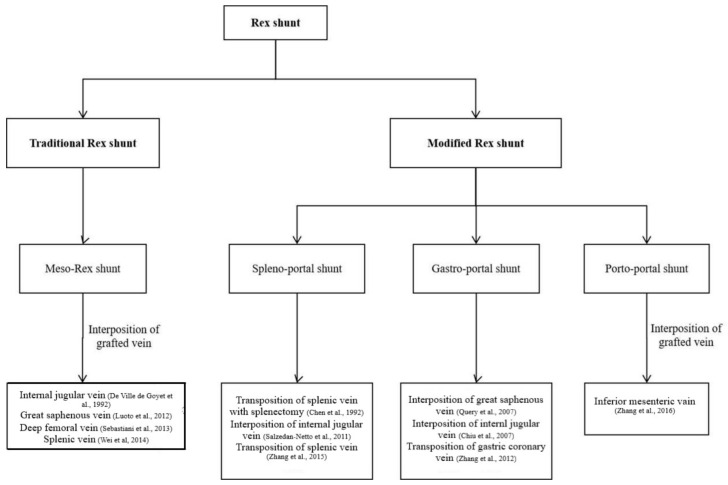
The various approaches of Rex shunt [3,4,5,6,7,8,9,10,11,12,13].

Surgical method of Rex shunt had experienced the modification of meso-Rex shunt, spleno-portal shunt, gastro-portal shunt, and porto-portal shunt since 1992 (Figure 2); however, its fundamental function is a reconstruction of the portal blood flow into the liver to restore the blood supply of the liver and relieve the extra-hepatic portal hypertension caused by EHPVO.

### 2.1. Meso-Rex Shunt

**Figure 3:**
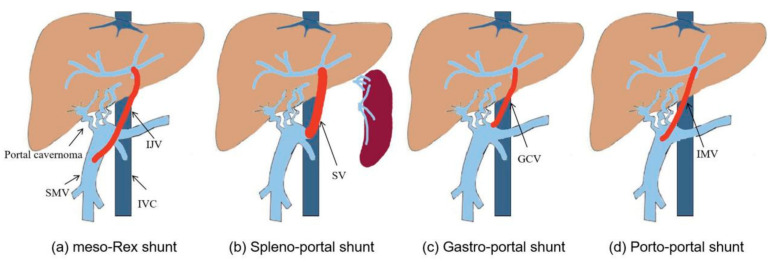
The Rex shunt including meso-Rex shunt (**a**), spleno-portal shunt (**b**), gastro-portal shunt (**c**) and porto-portal shunt (**d**) (IJV, internal jugular vein; SMV, superior mesenteric vein; IMV, inferior mesenteric vein; GCV, gastric coronary vein; SV, splenic vein; IVC, inferior vena cava).

In 1992, de Ville et al. [3] first used a superior mesenteric vein to left portal vein shunt with the interposition of an internal jugular vein to treat EHPVO patients after liver transplantation (Figure 3a), which successfully alleviated the symptoms of extra-hepatic portal hypertension by reconstructing the hepatopetal portal blood flow [14]. Since then, this operation became known as “meso-Rex shunt” or “Rex shunt” [15], which are also used to distinguish the traditional Rex shunt from the modified Rex shunt using another bypass vein. In the meso-Rex shunt, a great saphenous vein, deep femoral vein, or splenic vein can also be used as a grafted vein [4,5,6].

### 2.2. Spleno-Portal Shunt

The first application time of the modified Rex shunt was very close to that of the traditional Rex shunt (meso-Rex shunt). In 1992, a splenic vein to left portal vein shunt (spleno-portal shunt) was first performed by Chen et al. [7] to treat a child with EHPVO after splenectomy, during which a splenic vein was directly transposed to the Rex recessus and anastomosed with the left portal vein. This Rex shunt avoided the transplantation of the internal jugular vein in meso-Rex shunt, but this operation was only used to treat EHPVO children undergoing splenectomy, which may be one of the causes for its limited application. In order to avoid splenectomy, a spleen preserving spleno-portal shunt was used in 2011 by Netto et al. [8] to treat a child with the portal vein and superior mesenteric vein thrombosis. An internal jugular vein was interposed between the splenic vein and the left portal vein during this procedure. This child was not suitable for meso-Rex shunt due to the superior mesenteric vein thrombosis. Whilst this surgical approach provided a new option for treating the children with superior mesenteric vein thrombosis, it inevitably required neck surgery. In order to avoid splenectomy and neck surgery, we used a spleen preserving spleno-portal shunt in 2015 to treat four children with EHPVO [9]. During this approach, the proximal end of the splenic vein was directly transposed to the Rex recessus and anastomosed with the left portal vein (Figure 3b). However, the spleno-portal shunt is less frequently used due to its difficulty of exposing and dissecting the splenic vein.

### 2.3. Gastro-Portal Shunt

A gastric coronary vein to left portal vein shunt (gastro-portal shunt) is another modified Rex shunt. In 2007, Query et al. [10] used a gastro-portal shunt with the interposition of a great saphenous vein to treat a child with EHPVO, during which a great saphenous vein was interposed between the gastric coronary vein and left portal vein. In 2007, Chiu et al. [11] interposed an internal jugular veinbetween the gastric coronary vein and left portal vein to treat two children with EHPVO. Nevertheless, transplantation of a great saphenous vein or an internal jugular vein would require leg or neck dissection, which increases the surgical incision except for the abdominal incision to perform Rex shunt. Consequently, we used a gastro-portal shunt without a grafted vein to treat eight EHPVO children in 2012 [12]. In this approach, the gastric coronary vein was dissected proximally to the esophageal hiatus, and its distal end was transposed to the Rex recessus and anastomosed with the left portal vein (Figure 3c). This operation is widely used in treating EHPVO due to its reduction of vascular anastomotic number and avoidance of grafted veins in our center [16]. However, the gastro-portal shunt is not suitable for most EHPVO due to the requirement of the gastric coronary vein (no less than 5 mm in diameter and a suitable length) [16].

### 2.4. Porto-Portal Shunt

The inferior mesenteric vein is an important branch of the portal venous system that collects the blood of the left half of the colon, sigmoid colon, and rectum. Because there are many communicating venous arches, transection of the inferior mesenteric vein does not affect the blood supply of the colon and rectum. Based on this anatomy, the inferior mesenteric vein is a suitable graft. In 2003, Ates et al. [17] performed an inferior mesenteric vein to left portal vein shunt in order to treat a child with EHPVO, where the proximal end of the inferior mesenteric vein was transposed to the Rex recessus and anastomosed with the left portal vein, avoiding transplantation of other blood vessels and simplifying the operation. Nevertheless, the inferior mesenteric vein needs to cross the front of the pancreas and pass through the back of the stomach in its transposition to the Rex recessus, which may affect the patency of the bypass vein due to angulation and compression from surrounding tissue. In addition, the inferior mesenteric vein requires a sufficient length. In 2016, we used a portal cavernoma to left portal vein shunt with the interposition of the inferior mesenteric vein (porto-portal shunt) for the treatment of nine children with EHPVO [13], during which a grafted inferior mesenteric vein was interposed between the dilated part of portal cavernoma and the left portal vein (Figure 3d), thus reducing the length of grafted vein compared with the meso-Rex shunt and avoiding other surgical incisions. The porto-portal shunt is suitable for almost all EHPVO due to natural conditions of inferior mesenteric vein as a grafted vein, which was considered an optimal modified Rex shunt in our center [16].

## 3. The Indications of Rex Shunt

### 3.1. Surgical Indications

Due to its function of reconstructing portal blood flow into the liver, Rex shunt is often used to treat EHPVO caused by thrombosis and tumor embolism and can also be used for portal vein reconstruction during liver transplantation. In 2006, Superina et al. [2] formulated a surgical guideline of EHPVO, which identified the preconditions for Rex shunt, including the diagnosis of EHPVO by portal venography, patent left portal vein by intra-hepatic portal venography or surgical exploration and without liver diseases (liver fibrosis or cirrhosis). According to the surgical guidelines of EHPVO [2], Rex shunt should be timely performed when there is an absolute indication including medically/endoscopically refractory variceal hemorrhage, severe hypersplenism (platelet count < 10,000), recurrent complications including non-variceal hemorrhage or infections, symptomatic and medically refractory porto-systemic encephalopathy, hepato-pulmonary syndrome and porto-pulmonary hypertension. Rex shunt can be postponed when there is a relative indication including symptomatic splenomegaly, unacceptable activity restrictions because of splenomegaly, large varices and poor access to health care, neuro-cognitive testing suggestive of porto-systemic encephalopathy, portal biliopathy and unexplained failure to thrive or delay in sexual development. Performing a prophylactic Rex shunt is also a feasible option. A negative correlation was found between shunt blood flow and operative age [18], which suggested that delaying operative age did not result in a better prognosis. Therefore, when EHPVO is diagnosed, and the indications for Rex shunt are met, Rex shunt should be timely performed.

### 3.2. Pre- and Intra-Operative Assessment

The diagnosis of EHPVO should be identified before making the decision of performing Rex shunt. Commonly, the abdominal ultrasound (US) and enhanced computed tomography (CT) or magnetic resonance angiography (MRA) are used to evaluate the anatomy of portal venous system. Although US is a simple and non-invasive diagnostic method, the accuracy of US in diagnosing EHPVO decreases due to the interference of abdominal gas or the existence of a dilated collateral straight portal vein. Abdominal CT or MRA can improve the accuracy of EHPVO diagnosis [19]. Furthermore, a retrograde portal venography is a necessary radiological detection due to its gold standard for diagnosing EHPVO. Except for diagnosis of EHPVO, the purpose of pre-operative assessment is to visualize the intrahepatic portal and hepatic veins, superior mesenteric vein, splenic vein, renal veins, and inferior vena cava above and below the liver. Finally, hemotological tests, including liver function tests, routine blood test, and coagulation function, should be performed to exclude liver disease and identify hypercoagulable states [2].

An intra-operative portal venography should be performed for children without a retrograde portal venography before surgery [9,12,16]. During the operation, the intra- and extra-hepatic portal vein system are visualized to identify the diagnosis of EHPVO and the patency of left portal vein, which provides a reliable imaging basis for selecting a suitable bypass vein and avoiding an unnecessary operation [20]. Therefore, portal venography has an important role in the performance of Rex shunt. In addition, liver biopsy should be performed to exclude significant intrinsic liver disease. The following conditions need to be satisfied for the performance of a modified Rex shunt: the diameter of the grafted vein is no less than 5 mm, and the length of the grafted vein is suitable for a tension-free anastomosis [9,12,16].

The interdisciplinary care is also very important, as gastroenterologists, surgeons, and pediatricians should be involved in diagnosis and management of EHPVO.

## 4. The Strengths of Rex Shunt

Therapeutic options for EHPVO include surgical methods and radiological interventions. The surgical methods include devascularization, endoscopic esophagogastric vein ligation and sclerotherapy, portosystemic shunt, and Rex shunt.

### 4.1. Devascularization, Endoscopic Esophagogastric Vein Ligation, and Sclerotherapy

The devascularization and endoscopic esophagogastric vein ligation and sclerotherapy can immediately stop upper gastrointestinal bleeding. Yet, the recurrence of esophagogastric varices is inevitable due to portal hypertension, which results in a high recurrence rate. Therefore, the devascularization and endoscopic esophagogastric vein ligation and sclerotherapy are usually used as an emergency method for temporary hemostasis [21,22]. Although splenectomy can effectively relieve hypersplenism, it cannot improve portal hypertension. In addition, the incidence of infection and portal vein thrombosis after splenectomy in children significantly increases [23,24,25,26]. Therefore, this approach is not recommended for treating portal hypertension in children.

### 4.2. Portosystemic Shunt

Portocaval shunt, proximal splenorenal shunt, and mesocaval shunt as non-selective shunt have been widely used to treat portal hypertension [22], which create a direct communication between the portal system and the systemic circulation, and depending on the flow, a full diversion can be achieved, with a consequent fall in the portal pressure. The duration from undergoing a surgery to the average life span in children is significantly longer than that of adults. Therefore, except for the surgical effectiveness, the postoperative quality of life should be considered after surgery for children. Although the non-selective shunt can significantly reduce portal pressure, it is prone to hepatic encephalopathy and liver failure due to the reduction of portal blood flow into the liver, which will worsen the postoperative quality of life. Therefore, the non-selective shunt is not an ideal surgery for EHPVO in children.

Distal splenorenal shunt (DSRS or Warren operation) as a selective shunt, which preserves portal and mesenteric blood flow to the liver, have been used with success to treat bleeding varices and hypersplenism and has been shown to reduce postoperative encephalopathy with equivalent long-term mortality and rebleeding rates. However, it cannot reconstruct the portal blood flow into the liver, and liver injury is inevitable due to the decreased portal blood flow into the liver. Therefore, the DSRS should be done at the time of surgery if the meso-Rex shunt cannot be completed because of anatomical issues [2].

### 4.3. Radiological Interventions

Transjugular intrahepatic portosystemic stent-shunt (TIPSS), as an important interventional therapy, is effective for treating hepatic or post-hepatic portal hypertension due to its advantages of avoiding a major abdominal surgery intervention and preserving the abdominal intact for possible transplantation. However, experience of TIPSS in children remains limited to case reports because it is more difficult in small patients [27]. Furthermore, portal cavernoma remains a relative contra-indication for TIPSS, and the presence of cavernoma has been associated with a significantly high failure rate of TIPSS [28]. Therefore, children with portal cavernoma are considered for TIPSS only if the meso-Rex shunt is not technically feasible [29].

A percutaneous transhepatic angioplasty/stenting is another interventional treatment for EHPVO (thrombosed portal vein), during which the portal trunk is recanalized through the placement of stent or balloon dilation in the portal vein. However, complete stent recanalization of EHPVO was possible only in one out of five children with the placement of stent due to difficulty attributable to the small caliber of veins [30]. In addition, EHPVO differs from common idiopathic prehepatic portal hypertension in that the intrahepatic portal system is large enough in diameter to allow transhepatic access and intravascular maneuvers. Therefore, though intervention is feasible for the treatment of EHPVO, it is rarely used in the treatment of children with EHPVO.

### 4.4. Rex Shunt

In addition to reducing portal pressure, Rex shunt avoids the occurrence of liver dysfunction caused by the insufficient blood supply to the liver by reconstructing the blood flow into the liver. Compared with portosystemic shunt, Rex shunt could significantly improve upper gastrointestinal bleeding, thrombocytopenia, coagulation function, serum albumin level, and body weight [31], thus indicating that Rex shunt can improve liver metabolic function. The deficiency of anticoagulant factors (protein C, protein S, and antithrombin III) was closely related to the occurrence of deep venous thrombosis (including portal vein thrombosis). Rex shunt could alleviate the deficiency of protein C, protein S, and antithrombin III in EHPVO by restoring the hepatopetal blood flow to improve the coagulation function and prevent bypass vein thrombosis [32].

The quality of life in children with EHPVO may be affected by splenomegaly-related pain, growth retardation, and portal cavernoma cholangiopathy (PCC). In one previous study, 54% of EHPVO children suffered from growth retardation due to malabsorption, deprivation of hepatotropic factors, chronic anemia, and growth hormone resistance [33]. Yet, Rex shunt can improve the growth parameters due to restoring blood supply to the liver [34]. According to a previous study, 92% (66/72) children with EHPVO had PCC due to compression on the biliary tree from long-standing portal cavernoma in the biliary and peribiliary region, where 85% of children were asymptomatic, and 7% were symptomatic [35]. The liver biochemistry was completely normalized after Rex shunt in 2/8 children with symptomatic PCC due to its decompression of portal cavernoma through restoring the blood flow into the liver [36]. Therefore, Rex shunt can improve the quality of life in children with EHPVO.

Progressive deterioration of liver functions and ascites may develop with increasing age, prolonged disease duration, and portal biliopathy in EHPVO due to decreasing hepatopetal portal blood flow [37]. On the other hand, Rex shunt can improve liver dysfunction. Therefore, Rex shunt should be performed as soon as possible, as postponing the performance of Rex shunt is not a wise choice [1].

In addition, no differences were reported in shunt complications, mortality, or gastrointestinal bleeding after surgery between meso-Rex shunt and portosystemic shunt in a meta-analysis, which indicated that meso-Rex shunt did not increase shunt complications, mortality, or gastrointestinal bleeding after surgery [38].

## 5. Effectiveness of Rex Shunt

The use of Rex shunt has a long history of nearly 30 years. Nevertheless, the postoperative failure rate was reported to vary from 8% to 40% due to the differences in surgical techniques and postoperative management among medical centers. By summarizing the efficacy of Rex shunt reported by various centers (Table 1), it was found that the failure rate after Rex shunt was about 10–20%. The complications include obstruction, thrombus, and stenosis of shunt after Rex shunt, and 24.2% (16/66) of post-operative complication was reported by a meta-analysis review [38]. Although patients may die after Rex shunt, a meta-analysis review showed 0% of mortality after Rex shunt [38]. In addition, no one don’t want to undergo a treatment after suffering from portal hypertension, which may be the reason that there is no report about survival of EHPVO patients without any treatment. Consequently, the challenge of Rex shunt is how to improve the prognosis of Rex shunt through preventing post-operative complication.

## 6. Factors Affecting the Efficacy of Rex Shunt

The efficacy of Rex shunt was affected by many factors, including thrombosis and stenosis of the bypass vein, dysplasia of the left portal vein, and previous operation history. The shunt thrombosis and stenosis are the commonly complications that lead to surgical failure after Rex shunt.

### 6.1. Shunt Thrombosis

The incidence of shunt thrombosis after Rex shunt was about 4–14% [40,41]. It has been reported that postoperative anticoagulant therapy can reduce the incidence of shunt thrombosis [41]. Long-term oral anticoagulant drugs did not increase the incidence of upper gastrointestinal bleeding in EHPVO and were also beneficial to reduce the portal pressure in EHPVO with a high-coagulation state [44]. Especially, anticoagulant therapy can prevent thrombosis in children with abnormal coagulation mechanisms [45].

### 6.2. Shunt Stenosis

The incidence of shunt stenosis after Rex is about 1.6–17.4% [46,47]. Shunt stenosis often occurs at the anastomotic orifice in Rex recessus, which might be related to dysplasia of intrahepatic portal vein, hyperplasia of hepatic parenchyma, and anastomotic angulation [47]. The selection of grafted vein was related to the prognosis of Rex shunt. A comparative study on the efficacy of various modified Rex shunts in our center revealed that the postoperative recurrence rate of porto-portal shunt was 11.5%, which was significantly lower than 22.9% of gastro-portal shunt [16]. Following reasons may explain the different prognosis: (1) the gastric coronary vein was secondarily dilated after portal hypertension and thus could be used as a grafted vein due to its diameter that was no less than 5 mm. However, the lumen of the gastric coronary vein was narrowed when the portal pressure was reduced after Rex shunt, which might lead to shunt stenosis after Rex shunt. (2) The tortuous situation and many collateral veins of the gastric coronary vein might affect the patency of the bypass vein [16].

### 6.3. Dysplasia of the Left Portal Vein and Previous Operation History

The dysplasia of the left portal vein could be identified by intra-hepatic portal venography before operation. The failure of Rex shunt caused by the dysplasia of the left portal vein should be avoided through intra-hepatic portal venography. The success rate of Rex shunt was significantly reduced in patients who previously underwent abdominal surgery or splenectomy, which might be related to the vascular angulation and thrombosis caused by abdominal adhesion [48].

### 6.4. Risk Factors of Rex Shunt Failure

The prior portosystemic shunts or mesenteric embolizations, the selection of bypass vein, and the age at operation have a deleterious effect on outcome after Rex shunt, which could be considered as the risk factors of Rex shunt failure. The unsuccessful manipulation of the mesenteric venous system (including splenorenal shunt, mesocaval shunt and splenectomy, mesenteric embolization) before an attempted Rex shunt had a significant adverse effect on the ability to successful restore portal flow (success rate: 63.6% with a previous operation vs. 88.3% without a previous operation) [48]. The failure rate of gastro-portal shunt was significantly higher than that of porto-portal shunt (22.9% vs. 11.5%) [16], which suggested that a tortuous left gastric vein with abundant branches was not a suitable bypass vein. The ability of the portal venous system to adapt to restored flow was age-dependent after Rex shunt [18]. A significant inverse correlation between portal blood flow achieved and the age at operation suggests increasingly poor compliance of the intrahepatic venous system overtime, which could affect the outcomes after Rex shunt.

## 7. Methods That Improve the Prognosis of Rex Shunt

Based on the above-mentioned factors affecting the prognosis of Rex shunt, the following methods should be performed in order to improve its prognosis: (1) Identifying the surgical indications of Rex shunt [2]. (2) Selecting an appropriate grafted vein. The selection of the bypass vein is related to the success of Rex shunt. The grafted vein with a diameter of no less than 5 mm and a length with a tension-free anastomosis should be considered as a suitable bypass vein in Rex shunt [9,12,16]. When a gastric coronary vein is used as a bypass vein, the gastric coronary vein should be straight with fewer collateral branches [16]. (3) Anticoagulant therapy should be performed after Rex shunts, as it is beneficial to prevent post-operative bypass vein thrombosis. (4) Vascular anastomotic technique should be improved. Although there is no report that the vascular anastomotic technique is related to the prognosis of Rex shunt, it is undeniable that the technical ability of the operator is an important factor affecting the prognosis of Rex shunt.

## 8. Treatment Strategy for Recurrence after Rex Shunt

### 8.1. Post-Operative Monitoring

The surgical prognosis should be evaluated through abdominal US, CT, and routine blood test after Rex shunt, which involves the patency, diameter and blood flow velocity of shunt, splenic size, and the counts of white blood cells, red blood cells, and platelet. The post-operative monitoring is recommended at 1, 3, 6, and 12 months and every 6 months thereafter [16].

The recurrence after Rex shunt refers to the patients undergoing Rex shunt suffer from extra-hepatic portal hypertension after surgery, which includes the manifestation of upper gastrointestinal bleeding, splenomegaly, and hypersplenism [16]. The most significant manifestation of recurrence after Rex shunt is the refractory upper gastrointestinal bleeding, following which abdominal US and CT examination should be performed to identify shunt stenosis or thrombosis. For patients without upper gastrointestinal bleeding, the aggravation or non-remission of splenomegaly and thrombocytopenia should be considered as the warning manifestation possibly related to the recurrence after Rex shunt. Furthermore, abdominal US and CT should be performed to identify the situation of the bypass vein [47]. It is helpful to evaluate the patency of the bypass vein through comparison with the previous examination results. Generally, the diagnosis of shunt stenosis is made when the diameter of the bypass vein is less than 3 mm in US or CT [47]. Splenomegaly and repeated bleeding of esophageal and gastric varices are the secondary symptoms of shunt stenosis or thrombosis.

### 8.2. Interventional Treatment

**Figure 4:**
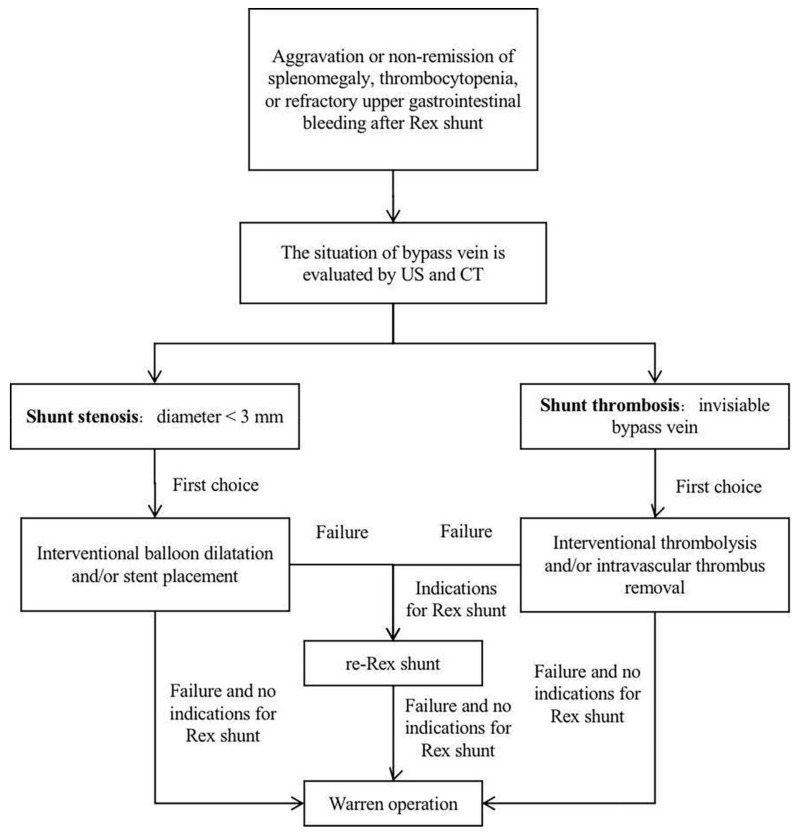
Treatment strategies for recurrence after Rex shunt.

The treatment of shunt thrombosis and stenosis after Rex shunt includes thrombolysis, balloon dilatation, stent placement, and shunt surgery [41,47,49]. Lautz et al. [47] treated 15 children with shunt stenosis after Rex shunt by the interventional balloon dilatation and stent placement, where 9 cases were successfully treated, 3 cases still had stenosis after the operation, 2 cases finally underwent a re-Rex shunt, and 1 case finally underwent a splenorenal shunt. Guerin et al. [42] reported nine cases of shunt stenosis or thrombosis after Rex shunt, where six cases underwent a re-operation (three cases underwent a portosystemic shunt, of which one case was successful; three cases underwent a re-Rex shunt, of which one case was successful), and three cases underwent the non-surgical treatment (two cases underwent an intravascular thrombus removal, and one case underwent an anticoagulant treatment). It can be seen that interventional balloon dilatation and stent placement could be successfully used to treat patients with shunt stenosis; however, surgical treatment should be further used for those who fail in the interventional treatment. For shunt thrombosis, interventional thrombolysis and intravascular thrombus removal can be firstly used; yet, surgery should be further used in the treatment of failed patients (Figure 4).

### 8.3. Surgical Treatment

Warren operation and Rex shunt are the main surgical methods for treating recurrence after Rex shunt. Bhat et al. [41] treated nine (14%, 9/65) children with shunt thrombosis after Rex shunt by a re-Rex shunt, six (6/9) of whom had shunt thrombosis after the re-Rex shunt; two (2/6) underwent interventional thrombolysis due to failure of the re-Rex shunt (one case was successfully treated with Rex shunt again), and four (4/6) were successfully treated with Warren operation, resulting in the re-Rex shunt failure rate of 66.7% (6/9). Luoto et al. [4] successfully treated two cases with shunt atresia after Rex shunt by Warren operation. Superina et al. [18] reported that two out of three patients with recurrence after Rex shunt were successfully treated by Warren operation. Zhang et al. [46] found that the failure rate of re-Rex shunt was 62.5% (5/8), which was significantly higher than 20% (1/5) of Warren operation in the treatment of recurrence after Rex shunt. However, Rex shunt should still be the first surgical treatment option due to its reconstruction of hepatopetal portal blood flow, and the Warren operation should be selected in the cases of failure in a re-Rex shunt [1] (Figure 4).

The treatment strategy shown by Figure 4 is based on the published literature, but it does not restrict the usage of other surgery, such as liver transplantation (LT), which is more physiologic than Warren operation and may help preserve autologous vessels in the treatment of patients with a failed re-Rex shunt. LT may be indicated in the rare patient with life-threatening complications of EHPVO not manageable by conservative management or other surgery. These complications include those with encephalopathy or porto-pulmonary syndrome (hypoxia and hepato-pulmonary syndrome or pulmonary arterial hypertension) who cannot benefit from Rex shunt [2,22].

## 9. Conclusions

Rex shunt has been widely used in the treatment of EHPVO in children and has been developed and modified through the meso-Rex shunt, spleno-portal shunt, gastro-portal shunt, and porto-portal shunt. However, there is still 8–40% of failure rate after Rex shunt due to shunt thrombosis and stenosis. Therefore, to improve its prognosis, surgical indications, an appropriate grafted vein, post-operative anticoagulant therapy, and vascular anastomotic technique should be assured. In addition, interventional and surgical treatment could be used for treatment of recurrence after Rex shunt.

## Figures and Tables

**Table 1:** The efficacy of Rex shunt reported by various centers.

Time of Publication	First Author	Medical Center (Country)	Time of Cases	Duration of Follow-Up (Median) (Months)	Number of Cases	Failure Rate (%)
2009	Krebs-Schmitt, D. [39]	University Medical Center, Hamburg (Germany)	2009	18–146 (109)	25	40 (10/25) IJV:12
2010	Sharif, K. [40]	Birmingham Children’s Hospital (UK)	1998–2003	63.6–105.6 (96)	29	8 (2/24)
2012	Luoto, T. [4]	Hospital for Children and Adolescents, University of Helsinki (Finland)	2002–2010	6–60	21	6 months: 0 1 year: 12 (2/16) 3 years: 17 (3/14) 5 years: 31 (4/13)
2013	Bhat, R. [41]	Ann and Robert H. Lurie Children’s Hospital of Chicago (USA)	1999–2009	0.07–111 (30.8)	65	14 (5/65)
2013	Guerin, F. [42]	Bicetre hospital (France)	1996–2010	6–167 (55)	32	18.8 (6/32)
2017	Zhang, J.S. [16]	Capital Institute of Pediatrics (China)	2008–2016	1–90 (20)	79	19 (15/79) PP: 11.5%
2020	Ruan, Z. [43]	The Second Hospital of Shandong University (China)	2010–2017	6	50	12 (6/50)

IVJ, meso-Rex shunt; PP, porto-portal shunt.

## Data Availability

Not applicable.

## References

[1] Shneider B.L., Bosch J., De Franchis R., Emre S.H., Groszmann R.J., Ling S.C., Lorenz J.M., Squires R.H., Superina R.A., Thompson A.E. (2012). Portal hypertension in children: Expert pediatric opinion on the report of the Baveno V Consensus Workshop on Methodology of Diagnosis and Therapy in Portal Hypertension. Pediatr. Transplant..

[2] Superina R., Shneider B., Emre S., Sarin S., Goyet J.D.V.D. (2006). Surgical guidelines for the management of extra-hepatic portal vein obstruction. Pediatr. Transplant..

[3] De Ville de Goyet J., Clapuyt P., Otte J.B. (1992). Extrahilar mesenterico-left portal shunt to relieve extrahepatic portal hypertension after partial liver transplant. Transplantation.

[4] Luoto T., Pakarinen M., Mattila I., Rintala R. (2012). Mesoportal bypass using a constructed saphenous vein graft for extrahepatic portal vein obstruction-technique, feasibility, and outcomes. J. Pediatr. Surg..

[5] Sebastiani S., Martens T., Randon C., De Jaeger A., De Bruyne R., Voet D., Troisi R. (2013). Meso-Rex shunt using deep femoral vein conduit: First report. Acta Chir. Belg..

[6] Wei Z., Rui S.G., Yuan Z., Guo L.D., Qian L., Wei L.S. (2014). Partial splenectomy and use of splenic vein as an autograft for meso-Rex Bypass: A clinical observational study. Med. Sci. Monit..

[7] Chen V.T.-K., Wei J., Liu Y.-C. (1992). A New Procedure for Management of Extrahepatic Portal Obstruction: Proximal splenic-left intrahepaic portal shunt. Arch. Surg..

[8] Salzedas-Netto A.A., Duarte A.A.B., Linhares M.M., Mattar R.H., Medeiros K.L., Cury E.K., Filho G.D.J.L., Gonzalez A.M., Martins J.L. (2011). Variation of the Rex shunt for treating concurrent obstruction of the portal and superior mesenteric veins. J. Pediatr. Surg..

[9] Zhang J.-S., Li L., Hou W.-Y., Liu S.-L., Diao M., Zhang J., Li Q., Ye M., Ming A.-X., Dong N. (2015). Spleen-preserving proximal splenic-left intrahepatic portal shunt for the treatment of extrahepatic portal hypertension in children. J. Pediatr. Surg..

[10] Query J.A., Sandler A., Sharp W.J. (2007). Use of autogenous saphenous vein as a conduit for mesenterico-left portal vein bypass. J. Pediatr. Surg..

[11] Chiu B., Pillai S.B., Sandler A.D., Superina R.A. (2007). Experience with alternate sources of venous inflow in the meso-Rex bypass operation: The coronary and splenic veins. J. Pediatr. Surg..

[12] Zhang J.-S., Li L., Liu S.-L., Cheng W., Diao M., Hou W.-Y., Zhang J., Li S.-L., Liu Y., Wang H.-B. (2012). Gastroportal shunt for portal hypertension in children. J. Pediatr. Surg..

[13] Zhang J.-S., Li L., Cheng W. (2016). A New Procedure for the Treatment of Extrahepatic Portal Hypertension in Children: Portal Cavernoma-Rex Shunt with Interposition of Grafted Portal Vessel. J. Am. Coll. Surg..

[14] De Goyet J.D.V., Alberti D., Falchetti D., Rigamonti W., Matricardi L., Clapuyt P., Otte J.B., Caccia G. (1999). Treatment of Extrahepatic Portal Hypertension in Children by Mesenteric-to-left Portal Vein Bypass: A New Physiological Procedure. Eur. J. Surg..

[15] Bambini D.A., Superina R., Almond P., Whitington P.F., Alonso E. (2000). Experience with the Rex shunt (mesenterico-left portal bypass) in children with extrahepatic portal hypertension. J. Pediatr. Surg..

[16] Zhang J.-S., Li L., Cheng W. (2017). The optimal procedure of modified Rex shunt for the treatment of extrahepatic portal hypertension in children. J. Vasc. Surg. Venous Lymphat. Disord..

[17] Ateş O., Hakgüder G., Olguner M., Akgür F.M. (2003). Extrahepatic portal hypertension treated by anastomosing inferior mesenteric vein to left portal vein at rex recessus. J. Pediatr. Surg..

[18] Superina R., Bambini D.A., Lokar J., Rigsby C., Whitington P.F. (2006). Correction of extrahepatic portal vein thrombosis by the mesenteric to left portal vein bypass. Ann. Surg..

[19] Gauthier F. (2005). Recent concepts regarding extra-hepatic portal hypertension. Semin. Pediatr. Surg..

[20] Zhang J.-S., Li L. (2020). Imaging features and clinical relevance of portal venous systems shown by extrahepatic portal angiography in children with extrahepatic portal venous obstruction. J. Vasc. Surg. Venous Lymphat. Disord..

[21] Sarma M.S., Seetharaman J. (2021). Pediatric non-cirrhotic portal hypertension: Endoscopic outcome and perspectives from developing nations. World J. Hepatol..

[22] De Ville de Goyet J., D’Ambrosio G., Grimaldi C. (2012). Surgical management of portal hypertension in children. Semin. Pediatr. Surg..

[23] Leonard A.S., Giebink G.S., Baesl T.J., Krivit W. (1980). The overwhelming postsplenectomy sepsis problem. World J. Surg..

[24] Kristinsson S.Y., Gridley G., Hoover R.N., Check D., Landgren O. (2013). Long-term risks after splenectomy among 8,149 cancer-free American veterans: A cohort study with up to 27 years follow-up. Haematologica.

[25] Boyer T.D., Habib S. (2014). Big spleens and hypersplenism: Fix it or forget it?. Liver Int..

[26] Machado N.O., Chopra P.J., Sankhla D. (2010). Portal vein thrombosis postlaparoscopic splenectomy presenting with infarction of gut: Review of risk factors, investigations, postoperative surveillance, and management. Surg. Laparosc. Endosc. Percutaneous Tech..

[27] Heyman M.B., LaBerge J.M., Somberg K.A., Rosenthal P., Mudge C., Ring E.J., Snyder J.D. (1997). Transjugular intrahepatic portosystemic shunts (TIPS) in children. J. Pediatr..

[28] Tripathi D., Stanley A.J., Hayes P.C., Travis S., Armstrong M.J., Tsochatzis E.A., Rowe I.A., Roslund N., Ireland H., Lomax M. (2020). Transjugular intrahepatic portosystemic stent-shunt in the management of portal hypertension. Gut.

[29] Di Giorgio A., Nicastro E., Agazzi R., Colusso M., D’Antiga L. (2020). Long-term Outcome of Transjugular Intrahepatic Portosystemic Shunt in Children with Portal Hypertension. J. Pediatr. Gastroenterol. Nutr..

[30] Cwikiel W., Keussen I., Larsson L., Solvig J., Kullendorff C.-M. (2003). Interventional treatment of children with portal hypertension secondary to portal vein occlusion. Eur. J. Pediatr. Surg..

[31] Lautz T., Keys L.A., Melvin J.C., Ito J., Superina R.A. (2013). Advantages of the meso-Rex bypass compared with portosystemic shunts in the management of extrahepatic portal vein obstruction in children. J. Am. Coll. Surg..

[32] Mack C.L., Superina R.A., Whitington P.F. (2003). Surgical restoration of portal flow corrects procoagulant and anticoagulant defi-ciencies associated with extrahepatic portal vein thrombosis. J. Pediatr..

[33] Sarin S.K., Bansal A., Sasan S., Nigam A. (1992). Portal-vein obstruction in children leads to growth retardation. Hepatology.

[34] Lautz T.B., Sundaram S.S., Whitington P.F., Keys L., Superina R.A. (2009). Growth impairment in children with extrahepatic portal vein obstruction is improved by mesenterico-left portal vein bypass. J. Pediatr. Surg..

[35] Sarma M.S., Yachha S.K., Rai P., Neyaz Z., Srivastava A., Poddar U. (2018). Cholangiopathy in children with extrahepatic portal venous obstruction. J. Hepato-Biliary-Pancreat. Sci..

[36] Gauthier-Villars M., Franchi S., Gauthier F., Fabre M., Pariente D., Bernard O. (2005). Cholestasis in children with portal vein obstruction. J. Pediatr..

[37] Rangari M., Gupta R., Jain M., Malhotra V., Sarin S. (2003). Hepatic dysfunction in patients with extrahepatic portal venous obstruction. Liver Int..

[38] Yamoto M., Chusilp S., Alganabi M., Sayed B.A., Pierro A. (2021). Meso-Rex bypass versus portosystemic shunt for the management of extrahepatic portal vein obstruction in children: Systematic review and meta-analysis. Pediatr. Surg. Int..

[39] Krebs-Schmitt D., Briem-Richter A., Grabhorn E., Burdelski M., Helmke K., Broering D.C., Ganschow R. (2009). Effectiveness of Rex shunt in children with portal hypertension following liver transplantation or with primary portal hypertension. Pediatr. Transplant..

[40] Sharif K., Mckiernan P., Goyet J.D.V.D. (2010). Mesoportal bypass for extrahepatic portal vein obstruction in children: Close to a cure for most!. J. Pediatr. Surg..

[41] Bhat R., Lautz T.B., Superina R.A., Liem R. (2013). Perioperative strategies and thrombophilia in children with extrahepatic portal vein obstruction undergoing the meso-rex bypass. J. Gastrointest. Surg..

[42] Guérin F., Bidault V., Gonzales E., Franchi-Abella S., De Lambert G., Branchereau S. (2013). Meso-Rex bypass for extrahepatic portal vein obstruction in children. Br. J. Surg..

[43] Ruan Z., Wu M., Shao C., Zhang Y., Zhang C., Zhang F., Zhao B. (2020). Effects of Rex-bypass shunt on the cavernous transformation of the portal vein in children: Evaluation by the color Doppler ultrasonography. Insights Imaging.

[44] Kitchens C.S., Weidner M.H., Lottenberg R. (2007). Chronic oral anticoagulant therapy for extrahepatic visceral thrombosis is safe. J. Thromb. Thrombolysis.

[45] Khanna R., Sarin S.K. (2014). Non-cirrhotic portal hypertension—Diagnosis and management. J. Hepatol..

[46] Zhang J.-S., Li L., Cheng W. (2018). Surgical treatment for rebleeding caused by bypass failure after Rex shunt: Re-Rex shunt or Warren shunt?. Pediatr. Surg. Int..

[47] Lautz T.B., Kim S.T., Donaldson J.S., Superina R.A. (2012). Outcomes of percutaneous interventions for managing stenosis after meso-Rex bypass for extrahepatic portal vein obstruction. J. Vasc. Interv. Radiol..

[48] Chin A.C., Thow F., Superina R.A. (2008). Previous portal hypertension surgery negatively affects results of mesenteric to left portal vein bypass. J. Pediatr. Surg..

[49] Ketelsen D., Warmann S.W., Schaefer J.F., Haber P., Fuchs J., Claussen C.D., Brechtel K. (2012). Percutaneous revascularization of reoccluded meso-Rex shunts in extrahepatic portal vein obstruction. J. Pediatr. Surg..

